# Elevated Fasting Blood Glucose Is Predictive of Poor Outcome in Non-Diabetic Stroke Patients: A Sub-Group Analysis of SMART

**DOI:** 10.1371/journal.pone.0160674

**Published:** 2016-08-05

**Authors:** Ming Yao, Jun Ni, Lixin Zhou, Bin Peng, Yicheng Zhu, Liying Cui, SMART investigators

**Affiliations:** 1 Department of Neurology, Peking Union Medical College Hospital, Peking Union Medical College and Chinese Academy of Medical Sciences, Beijing,100730, China; 2 Neuroscience center, Chinese Academy of Medical Sciences, Beijing, China; Massachusetts General Hospital, UNITED STATES

## Abstract

**Background:**

Although increasing evidence suggests that hyperglycemia following acute stroke adversely affects clinical outcome, whether the association between glycaemia and functional outcome varies between stroke patients with\without pre-diagnosed diabetes remains controversial. We aimed to investigate the relationship between the fasting blood glucose (FBG) and the 6-month functional outcome in a subgroup of SMART cohort and further to assess whether this association varied based on the status of pre-diagnosed diabetes.

**Methods:**

Data of 2862 patients with acute ischemic stroke (629 with pre-diagnosed diabetics) enrolled from SMART cohort were analyzed. Functional outcome at 6-month post-stroke was measured by modified Rankin Scale (mRS) and categorized as favorable (mRS:0–2) or poor (mRS:3–5). Binary logistic regression model, adjusting for age, gender, educational level, history of hypertension and stroke, baseline NIHSS and treatment group, was used in the whole cohort to evaluate the association between admission FBG and functional outcome. Stratified logistic regression analyses were further performed based on the presence/absence of pre-diabetes history.

**Results:**

In the whole cohort, multivariable logistical regression showed that poor functional outcome was associated with elevated FBG (OR1.21 (95%CI 1.07–1.37), p = 0.002), older age (OR1.64 (95% CI1.38–1.94), p<0.001), higher NIHSS (OR2.90 (95%CI 2.52–3.33), p<0.001) and hypertension (OR1.42 (95%CI 1.13–1.98), p = 0.04). Stratified logistical regression analysis showed that the association between FBG and functional outcome remained significant only in patients without pre-diagnosed diabetes (OR1.26 (95%CI 1.03–1.55), p = 0.023), but not in those with premorbid diagnosis of diabetes (p = 0.885).

**Conclusion:**

The present results demonstrate a significant association between elevated FBG after stroke and poor functional outcome in patients without pre-diagnosed diabetes, but not in diabetics. This finding confirms the importance of glycemic control during acute phase of ischemic stroke especially in patients without pre-diagnosed diabetes. Further investigation for developing optimal strategies to control blood glucose level in hyperglycemic setting is therefore of great importance.

**Trial Registration:**

ClinicalTrials.gov NCT00664846

## Introduction

Ischemic stroke is one of the most common causes of disability and is considered now as the 4^th^ leading cause of death [1]. Hyperglycaemia after acute ischemic stroke (AIS) is present at admission up to 60% of patients, not only in those with diabetes but also in a large proportion of non-diabetics[2–4].Increasing evidences suggest that hyperglycemia following AIS adversely affects clinical and morphological outcome. Post-stroke hyperglycaemia is associated with increased final infarct volume [2,5–7], higher risk of secondary hemorrhagic transformation [2,8], and reduced recanalization rates after intravenous thrombolysis [9,10]. Hyperglycemia on admission is also strongly predictive of poor clinical outcome and higher mortality [2,5–8,11,12]. Based on these observations, the importance and potential benefit of tight glycaemic control in acute phase of stroke has become a research hotspot recently. Unfortunately, findings from a recent Cochrane review of 11 clinical trials demonstrated that active glycemic control after AIS failed to show clinical benefit [13].

Of note, stroke patients with and without diabetes were pooled for analysis in most of the previous studies. Whether the association between hyperglycaemia and functional outcome after AIS varies between diabetics and non-diabetics remains controversial. Limited studies of relative small sample-size issued this topic have reported conflicting results, suggesting a worse outcome in hyperglycemia patients regardless of the prior history of diabetes [14] or a detrimental effect of hyperglycemia only in non-diabetic patients [15–18]. Meta-analysis by Capes et al also showed that stress hyperglycemia was significantly associated with an increased risk of mortality and poor functional outcome after stroke in patients without diabetes but not in those with diabetes [2].However, most of the previous studies were of relative small-sample size [14–17,19], and ischemic and hemorrhagic stroke were pooled for analysis in several of these studies [17,19].In the present study based on data collected in a large sample of AIS patients, we aimed to investigate the relationship between the fasting blood glucose (FBG) and the 6-month functional outcome in a subgroup of SMART cohort and further to assess whether this association varied based on the status of previously diagnosed diabetes.

## Materials and Methods

### Subjects

Data were obtained from SMART study, which is a large prospective nationwide multi-center and cluster-randomized controlled trial to assess the effectiveness of a guideline-based structured care program for secondary stroke prevention, as opposed to usual care treatment, in China. Complete design and main results of the SMART study has been detailed elsewhere [20,21]. In brief, measures of intervention in SMART group was in line with the current guidelines for the secondary prevention of AIS, comprised of pharmaceutical treatment, lifestyle modification and patient education, while the usual care group received only interventions chosen by their physicians based on their knowledge or experiences.Glucose levels were determined by plasma venous FBG drawn within 24 hours after admission. Patient’s educational level was divided as three levels as following: illiterate, 6th Grade or lower and 7th Grade or higher [22]. The severity of index AIS was assessed by the National Institutes of Health Stroke Scale (NIHSS). Functional outcome was measured by Modified Rankin Scale (mRS) at 6-month post-stroke, and categorized as favorable when the patient remained independent (score 0–2) or poor when he became dependent (score 3–5).

Among the 3821 subjects included in SMART, 99 with transient ischemic attack and then 302 without available data of FBG were excluded. Five hundred and fifty-eight patients were further excluded due to the lack of follow-up information on their functional outcome at 6 months, leaving a working sample of 2862 participants (629 with premorbid diagnosis of diabetes) for this subgroup analysis.No difference was observed for the baseline demorgrahic and clinical characteristics between the patients included and excluded (data not shown).

The SMART study was performed with the approval of the central ethics committee at the leading study center at Peking Union Medical College Hospital and of the ethics committees at all participating sites. The written informed consents were obtained from all participants or their legal surrogates.

### Definitions of cerebrovascular risk factors

Hypertension was defined as receiving medication for hypertension or blood pressure ≥ 140/90mmHg. Hyperlipidemia was defined as receiving cholesterol reducing agents or overnight fasting cholesterol level ≥200 mg/dL. Diabetes status was assigned on the basis of a history of diabetes (FBG≥7.0mmol/L, or 2-hour postprandial blood glucose ≥11.1mmol/L) or treatment with hypoglycemic agents. History of stroke was defined according to both self-report and medical records.

### Statistical methods

Statistical analyses were performed using SPSS version 19.0 for Windows (SPSS Inc.). All p values were two-tailed and criteria for significance were p<0.05.

Continuous variables are presented as mean±SD, and categorical variables as percentages.Logistic regression models were computed with dichotomized functional outcome and using the favorable outcome (mRS 0–2) as the reference category. FBG concentration was used as a continuous measure. Univariate logistic regression analysis was firstly performed in the whole cohort to detect the potential risk factor related to functional outcome. Multivariate logistic regression model was then used in the whole cohort to evaluate the association between FBG and functional outcome. Candidate covariates (age, gender, educational level, history of hypertension and stroke, baseline NIHSS and group of treatment) were those associated with functional outcome in univariate analysis (P<0.10). The following interaction terms (age*hypertension, FBG*hypertension) were also separately introduced into the model, indicating non-significant interactions (p = 0.468 and p = 0.622, respectively).

To further explain whether the relationship between FBG and functional outcome is independent of the status of prior history of diabetes, additional stratified logistical regression analyses were performed according to the presence/absence of diabetic history. In the corresponding model, the same dependent variable and covariate were included as above-mentioned.

## Results

Baseline demographic and clinical characteristics of the whole cohort and potential risk factors related to functional outcome in univariate analysis are summarized in Table 1. Univariate logistic analysis showed that elevated FBG was correlated with poor functional outcome at 6 months (p = 0.003) in the whole cohort. Other predictors of functional status include age (p<0.001), NIHSS score (p<0.001), presence of pre-diagnosed hypertension (p = 0.006) and diabetes (p<0.001), SMART group (p = 0.005), and educational level (p<0.001). Men were also found to have a lower risk than women of presenting poor outcome (p = 0.022). In contrast, functional outcome was not found related to hypercholesterolemia and prior history of stroke (both p>0.05).

**Table 1:** Demographics and potential risk factors related to functional outcome by univariable analysis in whole cohort.

	Whole cohort (n = 2862)	Subgroup with mRS≤ 2 (n = 2606)	Subgroup with mRS> 2 (n = 256)	mRS>2vsmRS≤2	
				OR(95%CI)	P
**Age (years), mean±SD**	61.48±11.62	61.00±11.59	66.33±10.74	1.64(1.42–1.88)	**<0.001**
**Male gender**	68.9	69.5	62.5	0.73(0.56–0.96)	**0.022**
**Hypertension**	64.6	63.8	72.5	1.50(1.12–2.00)	**0.006**
**Hypercholesterolemia**	17.2	17.2	17.2	1.00(0.70–1.43)	0.994
**Diabetes**	22.0	21	32.4	1.81(1.37–2.39)	**<0.001**
**Previous ischemic Stroke**	24.8	24.1	31.9	1.48(1.12–1.95)	**0.006**
**Education level**	** **	** **	** **		
illiterate	6.9	6.4	12.1	Rf	
6^th^ Grade or lower	21.2	20.8	26.1	0.67(0.42–1.06)	0.089
7^th^ Grade or higher	71.9	72.8	61.8	0.45(0.30–0.69)	**<0.001**
**Fasting blood glucose (mmol/L), mean±SD**	6.28±2.54	6.23±2.52	6.74±2.73	1.18(1.06–1.31)	**0.003**
**NIHSS at baseline, mean±SD**	4.68±4.21	4.19±3.73	9.69±5.38	2.81(2.47–3.19)	**<0.001**
**SMART group**	45.9	45.1	54.3	1.45(1.12–1.87)	**0.005**

All data are presented as percentage unless otherwise indicated. P values correspond to the relationship between each variable and the severity of functional outcome measured by mRS. In each univariable logistic regression model, the severity of functional outcome was considered as the dependent variable. For continuous variables, the OR estimates the association related to an increase of 1 SD. Abbreviations: mRS, modified Ranking Scale; CI, confidence interval; OR, odds ratio; SD, standard deviation.

To evaluate the relationship between functional outcome and FBG, multivariate logistic regression, adjusting for age, gender, educational level, baseline NIHSS score, group of treatment, history of ischemic stroke and hypertension, was performed in the whole cohort (Table 2). Poor functional outcome was found to be associated with elevated FBG (OR1.21 (95%CI 1.07–1.37), p = 0.002), older age (OR1.64 (95%CI 1.38–1.94), p<0.001), higher NIHSS score (OR2.90 (95%CI 2.52–3.33), p<0.001) and history of hypertension (OR1.42 (95%CI 1.13–1.98), p = 0.04).

**Table 2:** Potential risk factors related to six-month post-stroke functional outcome in whole cohort regardless of the status of premorbid diagnosis diabetes.

	Crude Distribution		mRS>2vsmRS≤2	
	mRS≤ 2(n = 2606)	mRS> 2(n = 256)	OR (95%CI)	P
**Age (years), mean±SD**	61.00±11.59	66.33±10.74	1.64(1.38–1.94)	**<0.001**
**Male gender**	69.5	62.5	1.11(0.79–1.56)	0.549
**Hypertension**	63.8	72.5	1.42(1.13–1.98)	**0.042**
**Previous ischemic Stroke**	24.1	31.9	1.21(0.87–1.69)	0.253
**Education level**	** **	** **		
illiterate	6.4	12.1	Rf	
6^th^ Grade or lower	20.8	26.1	0.69(0.40–1.21)	0.194
7^th^ Grade or higher	72.8	61.8	0.66(0.38–1.13)	0.126
**Fasting blood glucose (mmol/L), mean±SD**	6.23±2.52	6.74±2.73	1.21(1.07–1.37)	**0.002**
**NIHSS at baseline, mean±SD**	4.19±3.73	9.69±5.38	2.90(2.52–3.33)	**<0.001**
**SMART group**	45.1	54.3	1.19(0.88–1.61)	0.251

All data are presented as percentage unless otherwise indicated. P values correspond to the relationship between each variable and the severity of functional outcome measured by mRS. In each polytomous logistic regression model, the severity of functional outcome was considered as the dependent variable. For continuous variables, the OR estimates the association related to an increase of 1 SD. Abbreviations: mRS, modified Ranking Scale; CI, confidence interval; OR, odds ratio; SD, standard deviation.

Multivariate stratified logistic regression analyses were further performed according to the presence/absence of diabetic history (Table 3 and Table 4). In the corresponding models, the same dependent variable and covariate were included as above-mentioned. In both subgroups, functional outcome was also found to be strongly associated with age (OR1.55 (95%CI 1.28–1.88), p<0.001 in non-diabetics; OR1.79 (95%CI 1.25–2.55), p<0.001 in diabetics) and NIHSS score (OR1.55 (95%CI 1.28–1.88), p<0.001 in non-diabetics; OR1.79 (95%CI 1.25–2.55), p<0.001 in diabetics). Of note, an elevated FBG level was associated with an increased disability in the patients without pre-diagnosed diabetes (OR1.26 (95%CI 1.03–1.55), p = 0.023), but this was not observed among patients with prior diabetes.

**Table 3:** Potential risk factors related to 6-month post-stroke functional outcome in subgroups without pre-stroke diagnosis of diabetes.

	Crude Distribution		mRS>2 vs mRS≤2	
	mRS≤2 (n = 2060)	mRS>2 (n = 173)	OR (95%CI)	p
**Age (years), mean±SD**	61.00±11.97	65.74±10.76	1.55(1.28–1.88)	**<0.001 **
**Male gender**	70.4	62.4	1.02(0.68–1.54)	0.918
**Hypertension**	60.7	68.6	1.41(0.95–2.08)	0.089
**Previous ischemic Stroke**	22.2	25.6	0.99(0.65–1.52)	0.975
**Education level**				
illiterate	6.6	13.6	Ref.	
6^th^ Grade or lower	21.7	26.0	0.68(0.35–1.30)	0.244
7^th^ Grade or higher	71.7	60.4	0.64(0.34–1.20)	0.163
**Fasting blood glucose(mmol/L), mean±SD**	5.58±1.70	5.93±2.09	1.26(1.03–1.55)	**0.023 **
**NIHSS at baseline, mean±SD**	4.14±3.78	9.93±5.58	2.79(2.39–3.26)	**<0.001 **
**SMART group**	44.4	52.6	1.152(0.80–1.65)	0.444

All data are presented as percentage unless otherwise indicated. P values correspond to the relationship between each variable and the 6-month functional outcome after stroke. In each polytomous logistic regression model, the severity of functional outcome was considered as the dependent variable. For continuous variables, the OR estimates the association related to an increase of 1 SD. Abbreviations: mRS, modified Ranking Scale; CI, confidence interval; OR, odds ratio; SD, standard deviation.

**Table 4:** Potential risk factors related to 6-month post-stroke functional outcome in subgroups with pre-stroke diagnosis of diabetes.

	Crude Distribution		mRS>2 vs mRS≤2	
	mRS≤2 (n = 546)	mRS>2 (n = 83)	OR (95%CI)	p
**Age (years), mean±SD**	62.15±9.95	67.57±10.66	1.79(1.25–2.55)	**0.001 **
**Male gender**	65.9	62.7	1.44(0.78–2.65)	0.24
**Hypertension**	75.3	80.5	1.29(0.64–2.59)	0.47
**Previous stroke**	31.0	45.1	1.55(0.88–2.72)	0.13
**Education level**				
illiterate	5.7	8.8	Ref.	
6^th^ Grade or lower	17.1	26.2	0.70(0.23–2.17)	0.56
7^th^ Grade or higher	77.2	65.0	0.61(0.21–1.78)	0.37
**Fasting blood glucose(mmol/L), mean±SD**	8.70±3.43	8.42±3.13	1.02(0.82–1.26)	0.885
**NIHSS at baseline, mean±SD**	4.36±3.57	9.19±4.90	3.28(2.40–4.48)	**<0.001 **
**SMART group**	45.1	54.3	1.29(0.74–2.25)	0.364

All data are presented as percentageunless otherwise indicated. P values correspond to the relationship between each variable and the 6-month functional outcome after stroke. In each polytomous logistic regression model, the severity of functional outcome was considered as the dependent variable. For continuous variables, the OR estimates the association related to an increase of 1 SD. Abbreviations: mRS, modified Ranking Scale; CI, confidence interval; OR, odds ratio; SD, standard deviation.

## Discussion

The present study demonstrated that elevated FBG level after stroke was associated with the poor functional outcome, in agreement with those of previous studies that identified hyperglycemia as an important predictor of an increased risk of disability in AIS [7,8,12,18]. However, this deleterious effect of hyperglycemia in our cohort was limited to patients without a prior history of diabetes. These intriguing findings further confirm and expand on the observations from previous studies based on a relative smaller sample-size and some data mixed hemorrhagic and ischemic stroke together, which showed that the harmful effect of hyperglycemia in acute stroke seems to be stronger in non-diabetics than in diabetics [2,15–18,23,24]. A meta-analysis by Capes et al [2] demonstrated that hyperglycemia decreased functional independence among stroke patients without history of diabetes. The hyperglycemic non-diabetic stroke patients were found to present with a 3.28-fold higher risk of death compared with those of euglycemia [2]. In another study, the association between blood glucose and mortality was not statistically significant in stroke patients with diabetes but in those without such history [18]. Roquer et al also found that hyperglycemia was correlated with stroke severity and identified as a marker of poor prognosis in non-diabetic patients with acute stroke but not in diabetics especially with poor glucose control [24]. Altogether, these findings suggest that the magnitude of the negative impact of hyperglycaemia on functional outcome after AIS may differ according to the status of premorbid diagnosis of diabetes, and that elevated FBG level post-stroke could be better for predicting prognosis in patients without prior diabetes than in those with diabetes. It is thus suggested that strategy of treatment on the high BG level after ischemic stroke should be varied according to the patients with or without diabetes. Unfortunately, no beneficial effect of intensive glucose control on clinical outcome after AIS was detected by previous intervention studies [13,25,26]. An ongoing prospective randomized clinical trial with a large sample-size (SHINE) might offer optimal management strategy of hyperglycemia in AIS [27].

Although diabetic patients have worse recovery prospects after AIS than non-diabetics [28], the independent and inverse impact of admission FBG on the functional outcome was only observed in the subgroup of non-diabetic AIS patients but not in diabetics in our cohort and other previous studies [2,15–18]. This discrepancy might be due to that chronic exposure to elevated blood glucose concentrations may offset adverse metabolic effects occurring in AIS, which may influence clinical outcome in non-diabetics. A relative minor increase of glucose level might produce an obvious harmful metabolic effect in patients with non-diabetics, but this might not be occurred in diabetics. Findings from previous animal experiments have demonstrated that hyperglycemia plays a detrimental effect on the brain in pre-ischemic glucose dependent manner [29]. Noteworthy, all of the acute hyperglycemic rats with glucose level higher than 16mmol/L developed post-ischemic seizures [29], which were merely observed in about 40% of streptozotocin-induced chronic hyperglycemic rats even with a much higher average glucose concentration of 18mmol/L [30]. This further supports that the detrimental effect of glucose on brain damage after ischemia is more severe in acute hyperglycemia than in chronic hyperglycemia. Second, the acute but not chronic hyperglycemia was found to be related to a delayed fibrinolytic process [10]. Acute increase of blood glucose may lead to glycation of key regulatory protein involving the fibrinolysis process, produce inhibited fibrinolytic activity, and then promote a thrombophilic state, which might also contribute to its adverse effect in the acute phase of AIS [31,32]. Third, it could not be excluded that certain medications usually used in diabetics, such as hypoglycemic agents, antiplatelet agents and statins, might in some way reduce the deleterious metabolic changes in the ischemic brain and thus confer a protective effect.

The methodological strengths of this study include the multicenter prospective design of the whole SMART cohort and the large sample size, even though this subgroup analysis is retrospective. Our study also contains potential limitations. First, data for glucosylated hemoglobin A1c (HbA1C) were only available in a relative small percentage of our cohort, which makes it unlikely to identify patients with unknown pre-stroke diabetes and to further explore whether the association between functional outcome and admission glucose varies according to chronic glucose level. However, in thrombolyzed AIS patients, chronic hyperglycemia measured by HbA1c did not convey prospective value for clinical outcome [10]. Second, although diabetes is a predictor of poor functional outcome (data not shown), it was not considered in the multivariable logistic model due to the interaction effect between diabetes and FBG on the relationship between FBG and functional outcome.Stratified analysis based on the status of pre-existing history of diabetes was further performed. Finally, the percentage of lack of follow-up data on functional outcome was considerable. However, no difference was observed for the baseline demographic and clinical characteristics between the patients included and excluded in the analysis (data not shown).

In conclusion, the results of our present study revealed that an elevated fasting glucose level after stroke was associated with the poor functional outcome at 6-month. This negative effect of elevated glucose level was obvious only in patients without prior history of diabetes. These results suggest that high glucose levels during acute ischemic stroke should be carefully managed especially in those without pre-diagnosed diabetes. Further investigation for developing optimal strategies to control blood glucose level in hyperglycemic setting is therefore of great importance.

## Supporting Information

S1 File Institutions participating SMART study (with local lead investigators). (DOCX)
