# Supplementary material for: Elevated Fasting Blood Glucose Is Predictive of Poor Outcome in Non-Diabetic Stroke Patients: A Sub-Group Analysis of SMART
Source: PLoS One. 2016 Aug 5;11(8):e0160674. doi: 10.1371/journal.pone.0160674 (PMC4975495; doi:10.1371/journal.pone.0160674)
Supplement: S1 File — (DOCX) [file pone.0160674.s001.docx]

Appendix I:

Institutions participating SMART study (with local lead investigators)

Beijing Daxing District Hospital (LZ Zhao );Beijing Ji Shui Tan Hospital, (YH Sun);Beijing Shi JiTan Hospital (ML He);China PLA General Hospital（CQ Pu）;China Rehabilitation Research Center (T Zhang); Daping Hospital (HD Zhou);First Affiliated Hospital of Xinjiang Medical University（XN Zhang) ;General Hospital of the Navy (XK Qi);Guangzhou First Municipal People’s Hospital （MY Li）;Henan Provincial People’s Hospital (JW Zhang);Huashan Hospital Fudan University (Q Dong);Inner Monoglia Batou City Central Hospital (JF Zhang);Luhe Hospital of Tongzhou District (HS Du);Jilin University (J Wu); Nanjing General Hospital of Nanjing Military Command,(XF Liu);No 263 Hospital of PLA (JL Zhang);No 309 Hospital of PLA,(YP Chen);Peking Union Medical College Hospital（LY Cui）; Peking University Third Hospital (DS Fan) ;Pinggu County Hospital (GY Zhang); Qilu Hospital of Shangdong University (CZ Yan);Qinghai Provincial People’s Hospital (SZ Wu);Shanghai Changzheng Hospital (XY Chen); Second Affiliated Hospital of Zhejiang University College of Medicine (MP Ding) ;Southwest Hospital (SG Shi);The Affiliated Drumtower Hospital of Nanjing University Medical School (72, Y Xu);The Appurtenant Hospital of Chifeng University (QF Cui);The First Affiliated Hospital of China Medical Sciences University (CD Zhang);The First Affiliated Hospital of College of Medicine (JW Wang) ;The First Affiliated Hospital Sun Yat-Sen University (QS Zeng);The First Hospital of Harbin Medical University (WZ Wang );The First Hospital of Hebei Medical University (MW Wang); The General Hospital Under Tianjin Medical Sciences University（Y Cheng ) ;The Second Hosptital of Harbin Medical University (LM Zhang);The Second Hospital of Hebei Medical University（ZZ Li) ;The Second Hospital of Shandong University (W Shang);The Second Affiliated Hospital of Soochow University (YJ Cao);The Third Hospital of Hebei North University（SM Yue）; The Third Hospital of Hebei Medical University (JY Liu); The Third People’s Hospital of Dalian (JB Zhang);The Zhongnan Hospital of Wuhan University (JJ Zhang);Tiantan Hospital, Capital Medical University (YJ Wang) ;Tianjin Third Central Hospital,(ZZ Zhang) ;West China Center of Medical Sciences (D Zhou); Xiangya Hospital of Central South University (J Xia); Xuanwu Hospital, Capital Medical University (JP Jia); Yutian County Hospital (JC Wang)
